# Quantum Quantifiers for an Atom System Interacting with a Quantum Field Based on Pseudoharmonic Oscillator States

**DOI:** 10.3390/e20080607

**Published:** 2018-08-16

**Authors:** Bahaaudin Mohammadnoor Raffah, Kamal Berrada

**Affiliations:** 1 Department of Physics, Faculty of Sciences, King Abdulaziz University, Jeddah 21589, Saudi Arabia; 2 Department of Physics, College of Science, Al Imam Mohammad Ibn Saud Islamic University (IMSIU), Riyadh 11623, Saudi Arabia

**Keywords:** pseudoharmonic oscillators, entanglement, von Neumann entropy, geometric phase, nonclassicality, squeezing entropies, quantum dynamics, photon-added coherent states

## Abstract

We develop a useful model considering an atom-field system interaction in the framework of pseudoharmonic oscillators. We examine qualitatively the different physical quantities for a two-level atom (TLA) system interacting with a quantized coherent field in the context of photon-added coherent states of pseudoharmonic oscillators. Using these coherent states, we solve the model that exhibits the interaction between the TLA and field associated with these kinds of potentials. We analyze the temporal evolution of the entanglement, statistical properties, geometric phase and squeezing entropies. Finally, we show the relationship between the physical quantities and their dynamics in terms of the physical parameters.

## 1. Introduction

Recently, the outgrowth and development of quantum information processing (QIP) have been supplied to enhance a large knowledge-base and increase the literature background of the quantum entanglement phenomenon, which is responsible for the implantation of the most tasks of QIP [1,2,3,4]. The importance of quantum entanglement in various applications of QIP has led to the examination and realization of high-dimensional systems and provided the significance of this kind of correlation in many-body quantum systems [5]. In recent years, various optical devices have been suggested to realize and generate the quantum entanglement, such as NMR systems [6], beam splitters [7], nanoresonators [8] and cavity QED [9]. Moreover, the generation of this kind of correlation actually emerges as an objective in the quantum experimental implementation when examining the non-classicality effects in quantum mechanics. Several attempts have been made to measure the quantum entanglement among particles and fields. Entanglement between atoms and photons has been treated and examined at optical frequencies with atoms [10] and electron spins [11], to interface stationary and flying qubits [12], to perform quantum communication [13] and to implement nodes for quantum repeaters [14] and networks [15].

The geometric phase (GP) is an example of the features of quantum mechanics that could remain overlooked by almost two generations of physicists. A considerable understanding of the formal description of quantum mechanics has been achieved after Berry’s discovery [16,17,18,19,20] of a geometric feature related to the dynamics of a quantum system in the adiabatic and cyclic unitary evolution of non-degenerate states. There are plenty of generalizations including nonadiabatic [17], non-cyclic and even nonunitary evolution of the quantum state. Berry has demonstrated that the wave function of a quantum system retains a memory of its evolution in its complex phase argument, which apart from the usual dynamical contribution, only depends on the geometry of the path traversed by the system. Known as the GP factor, this contribution originates from the very heart of the structure of quantum mechanics. The GP is attractive for the implementations of fault-tolerant quantum computation [21,22,23,24,25]. The idea is to exploit this inherent robustness provided by the topological properties of some quantum systems as a means of constructing built-in fault-tolerant quantum logic gates.

Squeezed states in quantized electromagnetic fields have attracted much attention and exhibited in several interesting works in the literature [26]. This squeezing physical concept has been extended to atomic systems [27] considering the definition used for the radiation field. In this context, the atomic squeezing has obtained a great deal of interest and provided many potential applications [28,29,30]. The atom-photon interactions are utilized to describe the conditions under which the squeezing effect will exist [31]. The appearance of atomic squeezing in a system of three-level atoms placed in a two-mode cavity is analyzed through the effective dipole-dipole interaction between atoms [32]. The model of spin-squeezed atoms, which is based on the Raman scattering with a strong laser pulse, was used to determine the transfer of the change of the correlation between the atom and the light [33]. Atomic squeezing under collective emission was considered to introduce a method for controlling the temporal behavior of the squeezing factor and characterizing the collective emission by the influence of the squeezing effect [34]. The squeezing effect in optimal and nonlinear spin states has been examined in [35,36], respectively. The relationship between the atomic spin squeezing and bosonic quadrature was introduced [37]. Moreover, the experimental realization for an ensemble of V-type atoms was reported [38,39]. In all these cases, the atomic squeezing has been treated in the framework of the Heisenberg uncertainty relations.

The Jaynes–Cummings (JC) model has received much interest, and various axes in different branches of the optical physics both theoretically and experimentally have been developed. The JC model has seen its real practice by exploiting the experimental step in the electrodynamics cavities. In order to understand the physical phenomena through that model, it is important to include the external noises on the studied quantum system [40,41,42,43]. Interestingly, it is shown that the noises that lead to the loss of energy have a significant impact on experimental progress in realistic physical situations. On the other hand, the noises that lead to destruction of the coherence in the system state also play a crucial role in those fields.

Coherent states play a crucial role in various physical branches [44,45], which are introduced as an eigenvector of the lowering operator for quantum harmonic oscillators [46]. These states exhibit physical properties like the classical electromagnetic field. In this context, the classical trajectory is used to determine the center of the coherent states’ wave packet for the harmonic oscillator potentials. There are other coherent states for nonlinear quantum electromagnetic fields, called nonclassical states, which are antibunching and sub-Poissonian statistics, squeezing and high order squeezing [47,48]. When the nonclassical quantum effects are taken into account, the classical limit and nonclassical limit of the radiation fields are determined by the ordinary coherent state.

The pseudoharmonic oscillator (PHO) potentials have attracted much attention, and more insights are being obtained on different physical subjects [49]. The PHO can be considered in a certain sense as an intermediate potential between the harmonic oscillator (HO) potential (an ideal potential) and anharmonic potentials (the more realistic potentials) [50]. A comparative analysis of potentials considering three-dimensional harmonic oscillator potential (HO-3D) and PHO was introduced in [51]. It is claimed that like the coherent states (CSs) for the HO, the CSs for the PHO can be helpful in the theory of quantum information [50]. In this context, it is shown that even if the HO-3D can be considered as a limit oscillator of the PHO, it is possible to find a harmonic limit that leads the obtained formulae for the PHO in the CSs formalism to the corresponding well-known formulae for the Glauber coherent state of the HO-1D (referring to a coherent state of a quantum simple harmonic oscillator) [52,53]. In fact, it is shown that apart from their theoretical merit (by contributing to a better understanding of the behavior and properties of the PHO), the formalism of the CSs of the PHO may have also a practical importance (by using it in the quantum information theory and practice) [50].

In the present manuscript, we consider the coherent states associated with the pseudoharmonic oscillator potentials and propose a new model of the atom-field in the framework of these kinds of potentials. We investigate the dynamical behavior of the atomic inversion, photons’ distribution, geometric phase, degree of entanglement and atomic squeezing for the quantum system, which will be described in the next section. The paper is organized as follows: In Section 2, we describe our Hamiltonian model and provide an exact form for the ket state of the system using the Schrödinger picture. Section 3 describes the quantum quantifiers considered in this manuscript. In Section 4, we show the numerical results and discuss the variation of the population inversion, entanglement, geometric phase and atomic squeezing. Finally, some conclusions are given in Section 5.

## 2. Physics Model and Dynamics

The PHO is considered as an anharmonic potential [51,54], which plays a similar role as the HO potential, and it admits exact mathematical studies. The PHO potential can be utilized in some cases as an intermediate oscillator between the HO and more anharmonic oscillators, e.g., Morse oscillator [55,56], Pöschl–Teller oscillator [57], which are more realistic. Similarly to the HO-1D (and a few other quantum systems, e.g., the Morse potential, as well as Poschl–Teller), the PHO potential accepts the building of coherent states [49,58]. Generally, the coherent states are of special importance due to their remarkable mathematical properties and interesting physical applications, especially in quantum optics [59] and also in quantum information theory [60]. The excitation on CSs can be considered as one of the possible generalizations of CSs. These states may be useful in the optical communications field, which employes the nonclassical signal beams, usually mixed with thermal noise [61]. On the other hand, the statistical properties of the CSs are useful in quantum optics and quantum electronics. A new class of states has been introduced, which are generated by the successive action of the raising operator on the Klauder–Perelomov coherent states of the PHO, and we have shown the important nonclassical properties such states possess [61].

The PHO effective potential has the form [58]:

(1) $$V\left(r\right)=\frac{M\omega^{2}}{8}r_{j}^{2}{\left(\frac{r}{r_{j}}-\frac{r_{j}}{r}\right)}^{2}+\frac{M\omega^{2}}{4}\left(r_{j}^{2}-r_{o}^{2}\right),$$

where ω presents the angular frequency, *M* defines the reduced mass and $r_{j}$ is the equilibrium distance depending on the rotational quantum *j*; the parameter α is given by:

(2) $$r_{j}=\left\{\frac{2\hbar }{M\omega }\sqrt{\alpha^{2}-\frac{1}{4}}\right\},\;\alpha =\sqrt{{\left(j+\frac{1}{2}\right)}^{2}+{\left(\frac{M\omega }{2\hbar }r_{0}^{2}\right)}^{2}}.$$

It is shown that the bounded states for the PHO are associated with the dynamical SU(1,1) group [49]. The su(1,1) Lie algebra is of great interest in quantum optics because it can characterize many kinds of quantum optical systems [62,63]. It has recently been utilized for investigating the nonclassical properties of light in quantum optical systems [45]. In particular, the bosonic realization of su(1,1) describes the degenerate and non-degenerate parametric amplifiers [64]. The squeezed states and nonlinear CSs of photons have been considered in terms of the su(1,1) Lie algebra and the CSs associated with this algebra [64]. The photon-added coherent states of the pseudoharmonic oscillator (PA-PHOCSs) are expanded as [65]:

(3) $$|z,m,k\rangle =\frac{1}{\sqrt{C_{k}\left({\left|z\right|}^{2}\right)}}\sum_{n=0}^{\infty }\frac{z^{n}}{\sqrt{R(n,k,m)}}|n+m,k\rangle ,$$

where:

(4) $$R\left(n,k,m\right)=\frac{\Gamma \left(2k\right){\left\{\Gamma (n+1)\right\}}^{2}}{\Gamma (n+m+2k)\Gamma (n+m+1)},$$

and:

(5) $$C_{k}\left({\left|z\right|}^{2}\right)=\sum_{n=0}^{\infty }\frac{z^{2n}}{R(n,k,m)}.$$

where *m* denotes the excited or the number of added photons and *k* is the Bragmann index. The energy spectrum of the PHO is identical to the HO-1D energy spectrum, up to a translation in the energy scale. It was demonstrated [66,67] that two specific elements of the positive discrete series of the SU(1,1) group unitary irreducible representations (for k=1/4 and k=3/4) reduce the corresponding representation spaces to the Hilbert space of the HO-1D.

The JC model is considered as one of the simplest models to describe the interaction between matter and radiation [68]. This model provides a considerably richer way to investigate the dynamical behavior of the physical phenomena that occur in the atom-field systems. In the rotating wave approximation limit, the model allows an explicit solution, which may be proven empirically. Here, we consider a TLA system interacting with PA-PHOCSs where the coupling term is dependent on the time,

(6) $$H_{I}\left(t\right)=\lambda \left(t\right)\left(\hat{a}\sqrt{{\hat{a}}^{†}\hat{a}}|0\rangle \langle 1|+\sqrt{{\hat{a}}^{†}\hat{a}}{\hat{a}}^{†}|1\rangle \langle 0|\right),$$

where $|1\rangle$ (respectively $|0\rangle$) defines the lower (respectively upper) level of the two-level atom (TLA), $\hat{a}$ (two-level atom ${\hat{a}}^{†}$) is correspondent to the annihilation (respectively creation) operator of the quantum field and $\lambda \left(t\right)=g\sin^{2}\left(t\right)$ is the coupling parameter, where in the case of constant coupling between the TLA and the field, it can be obtained at λ(t)=g. The time-dependent coupling λ(t) is assumed to be a sine function. In this context, the transient regime of the coupling varies rapidly with time. The generalization from the constant coupling λ to arbitrary time-dependent coupling λ(t) gives the possibility to model various new physical situations not discussed before. A realization of particular interest is when λ(t) may be the time-dependent alignment or orientation of the atomic/molecular dipole moment using a laser pulse [69] and the motion of the atom through the cavity. Theoretical examination of a cavity-quantum electrodynamics (QED) system monitored by utilizing bichromatic adiabatic passage under the influence of a dissipative environment [70], where the authors have analyzed the generation of a controlled Fock number state inside the cavity by a traveling atom, encounters the time-dependent effect and the delays of the Rabi frequencies of the laser fields and cavity.

We suppose that the TLA starts from the upper state $|0\rangle$, and the quantum field is prepared in the PA-PHOCS, $|z,m,k\rangle$; hence, the quantum state of the combined system is written as:

(7) $$\left|\varpi \left(0\right)\rangle =\right|\varpi_{A}\left(0\right)\rangle ⊗|z,m,k\rangle .$$

The ket state vector for any later time is written as:

(8) $$|\varpi (t)\rangle =\exp\left\{-i\int_{0}^{t}H_{I}\left(T\right)dT\right\}|\varpi \left(0\right)\rangle =\sum_{n=0}^{\infty }\left\{X_{n}\left(t\right)|n,u\rangle +Y_{n}\left(t\right)|n+1,l\rangle \right\}.$$

The time-dependent functions $X_{n}$ and $Y_{n}$ are given by:

$$\begin{array}{ccc} X_{n}\left(t\right) & = & Q_{n}\cos\left(f\left(t\right)\left(n+m+1\right)\right)\\ Y_{n}\left(t\right) & = & Q_{n}\sin\left(f\left(t\right)\left(n+m+1\right)\right), \end{array}$$

where |z,m,k〉 in Equation (7) can be written as:

$$|z,m,k\rangle =\sum_{n=0}^{\infty }Q_{n}|n\rangle ,$$

and:

$$f\left(t\right)=\left\{\begin{array}{cc} gt\;\; & for\;\;\;\;\lambda (t)=g\\ g\left(\frac{t}{2}-\frac{\sin(2t)}{4}\right)\;\; & for\;\;\;\lambda \left(t\right)=g\sin^{2}\left(t\right). \end{array}\right.$$

Once the wave function has been analytically obtained, it can be employed to analyze and discuss many physical features of the whole system and subsystems.

## 3. Quantum Quantifiers

In this section, we define and give a brief discussion of the different physical quantities. The atomic inversion is introduced as the probability difference of getting the TLA system in the upper and lower levels:

(9) $$S_{z}\left(t\right)=\sum_{n=0}^{\infty }\left\{{\left|X_{n}\left(t\right)\right|}^{2}-{\left|Y_{n}\left(t\right)\right|}^{2}\right\}.$$

When the field is defined in a Glauber state at t=0, the atomic inversion exhibits a collapse-revival feature during the time-evolution [71]. The origin of this phenomenon is dependent on the photon distribution of the field, and it is experimentally realizable through ionization detectors as the atomic beam leaving the cavity [72].

To examine the dynamical behavior of the entanglement for the TLA-field state, we introduce the von Neumann entropy as a measure, which is defined as [73]:

(10) $$S_{A}\left(t\right)=-Tr\left\{\rho_{A}\ln\rho_{A}\right\}=-\sum_{j=1}^{2}\mu_{j}\ln\mu_{j},$$

where $\rho_{A}$ (respectively $\rho_{F}$) presents the TLA (respectively field) density operator, obtained by making the trace over the quantum field (respectively TLA) element basis, i.e., $\rho_{A}={\mathrm{Tr}}_{F}\left(|\varpi (t)\rangle \langle \varpi (t)|\right)$) and $\mu_{j}$ denotes the eigenvalues of the TLA (respectively field) density operator. This entropy function changes from zero value for a factorizable state to one for a maximally-entangled state.

In order to analyze the photons’ distribution, we utilize Mandel’s parameter, which is considered as an accurate measure for the statistical properties of the quantum field. It is defined in terms of the average photon number of the field state as [74,75]:

(11) $$M_{P}=\frac{\langle N^{2}\rangle -{\langle N\rangle }^{2}-\langle N\rangle }{\langle N\rangle },$$

where:

(12) $$\langle N^{i}\rangle =\sum_{n=0}^{\infty }\left\{\frac{}{}n^{i}{\left|X_{n}\left(t\right)\right|}^{2}+{\left(n+1\right)}^{i}{\left|Y_{n}\left(t\right)\right|}^{2}\right\},\;\;\;\;i=1,2.$$

The $M_{P}$ parameter determines the statistical properties of the field state, where (−1≤$M_{P}<$ 0) corresponds to the sub-Poissonian photon distribution, $M_{P}>0$ is for super-Poissonian distribution and $Q_{P}=0$ is for the Poissonian distribution (semi-classical states).

The evolution of the quantum system is described as noncyclic, when the initial and final states are considered different. The initial and the final vector ket states are not connected through a complex scalar factor. If we assume that the initial ket state $|\varpi (0)\rangle$ evolves to $|\varpi (t)\rangle$ and the scalar product $M\left(t\right)=\langle \varpi (0)|\varpi (t)\rangle$ is expressed by a real number *ℓ*, where $M\left(t\right)=Re^{i\phi }$, consequently, the noncyclic phase is given by the angle ϕ. The cyclic geometric phase is considered as a particular case of the noncyclic phase, and it can be obtained by taking R=1. The Pancharatnam phase includes the geometric phase (GP) and dynamical phase and is defined as [76]:

(13) $$\Phi_{G}\left(t\right)=\arg\left(\langle \varpi (0)|\varpi (t)\rangle \right).$$

The Heisenberg uncertainty relation (HUR) is introduced to examine the squeezing entropy, which is described by Pauli matrices $\sigma_{x}$, $\sigma_{y}$ and $\sigma_{z}$ for the TLA in the framework of the quantum field as:

(14) $$\Delta \sigma_{x}\Delta \sigma_{y}\geq \frac{1}{2}\left|\langle \sigma_{z}\rangle \right|,$$

where $\Delta \sigma_{\alpha }=\sqrt{\langle \sigma_{\alpha }^{2}\rangle -{\langle \sigma_{\alpha }\rangle }^{2}}$. If $\sigma_{\alpha }$ verifies the condition:

(15) $$V\left(\sigma_{\alpha }\right)=\Delta \sigma_{\alpha }-\sqrt{\frac{\left|\langle \sigma_{z}\rangle \right|}{2}}<0,\;\alpha =x,y.$$

then, the atomic dipole fluctuation in $\sigma_{\alpha }$ will be squeezed.

For sets of complementary observable in an even-dimensional Hilbert space, an optimal entropic uncertainty relation has been studied through the quantum entropy theory [77],

(16) $$\sum_{k=1}^{N+1}H\left(\sigma_{k}\right)\geq \frac{N}{2}\ln\left(\frac{N}{2}\right)+\left(1+\frac{N}{2}\right)\ln\left(1+\frac{N}{2}\right),$$

with $H(\sigma_{k})$ giving the information entropy corresponding to the variable $S_{k}.$ For the general criterion of the squeezing, we employ entropic uncertainty relation (EUR) defined in Equation (16) in terms of the information entropy to examine the squeezing for the considered JC model. For the TLA state, $\rho_{A}$, the information entropies corresponding to the operators $\sigma_{x}$, $\sigma_{y}$ and $\sigma_{z}$ are given by:

(17) $$\begin{array}{ccc} H(\sigma_{x}) & = & -\left\{\frac{1}{2}+ℜ\left[\rho_{lu}\left(t\right)\right]\right\}\ln\left\{\frac{1}{2}+ℜ\left[\rho_{lu}\left(t\right)\right]\right\}\\ & & -\left\{\frac{1}{2}-ℜ\left[\rho_{lu}\left(t\right)\right]\right\}\ln\left\{\frac{1}{2}-ℜ\left[\rho_{lu}\left(t\right)\right]\right\}, \end{array}$$

(18) $$\begin{array}{ccc} H(\sigma_{y}) & = & -\left\{\frac{1}{2}+ℑ\left[\rho_{lu}\left(t\right)\right]\right\}\ln\left\{\frac{1}{2}+ℑ\left[\rho_{lu}\left(t\right)\right]\right\}\\ & & -\left\{\frac{1}{2}-ℑ\left[\rho_{lu}\left(t\right)\right]\right\}\ln\left\{\frac{1}{2}-ℑ\left[\rho_{lu}\left(t\right)\right]\right\}, \end{array}$$

(19) $$H\left(\sigma_{z}\right)=-\rho_{Uu}\left(t\right)\ln\rho_{Uu}\left(t\right)-\rho_{ll}\left(t\right)\ln\rho_{ll}\left(t\right),$$

where $\rho_{Uu}\left(t\right)=\sum_{n=0}^{\infty }{\left|X_{n}\left(t\right)\right|}^{2}$, $\rho_{ll}\left(t\right)=\sum_{n=0}^{\infty }{\left|Y_{n}\left(t\right)\right|}^{2}$ and $\rho_{lu}\left(t\right)=\sum_{n=0}^{\infty }X_{n}\left(t\right)Y_{n}^{*}\left(t\right)$. For a TLA N=2, then $0\leq H(\sigma_{\alpha })\leq \ln2$, while from Equation (16), we obtain that the information entropies corresponding to the operators $\sigma_{x},\sigma_{y}$ and $\sigma_{z}$ verify:

(20) $$H\left(\sigma_{x}\right)+H\left(\sigma_{y}\right)\geq 2\ln2-H\left(\sigma_{z}\right).$$

The aforementioned inequality may be also given as:

(21) $$\delta H\left(\sigma_{x}\right)\delta H\left(\sigma_{y}\right)\geq \frac{4}{\delta |H(\sigma_{z})|},$$

where:

(22) $$\delta H\left(\sigma_{\alpha }\right)=\exp\left[H\left(\sigma_{\alpha }\right)\right].$$

The EUR, described by Equation (21), evidences the impossibility of knowledge of simultaneous information about the observables $\sigma_{x}$ and $\sigma_{y},$ where the uncertainty of the polarization component $\sigma_{x}$ (respectively $\sigma_{y}$) is measured by $\delta H(\sigma_{x})$ (respectively $\delta H(\sigma_{y})$).

Let us now introduce the squeezing of the TLA using EUR defined in Equation (21), which is called squeezing entropy [77]. The component fluctuations $\sigma_{\alpha }$ (α=x or *y*) of the TLA are said to be squeezed if the entropy $H(\sigma_{\alpha })$ of $\sigma_{\alpha }$ verifies the inequality,

(23) $$E\left(\sigma_{\alpha }\right)=\delta H\left(\sigma_{\alpha }\right)-\frac{2}{\sqrt{\left|\delta H(\sigma_{z})\right|}}<0,\;\;\alpha =x,y.$$

## 4. Numerical Results and Discussion

**Figure 1:**
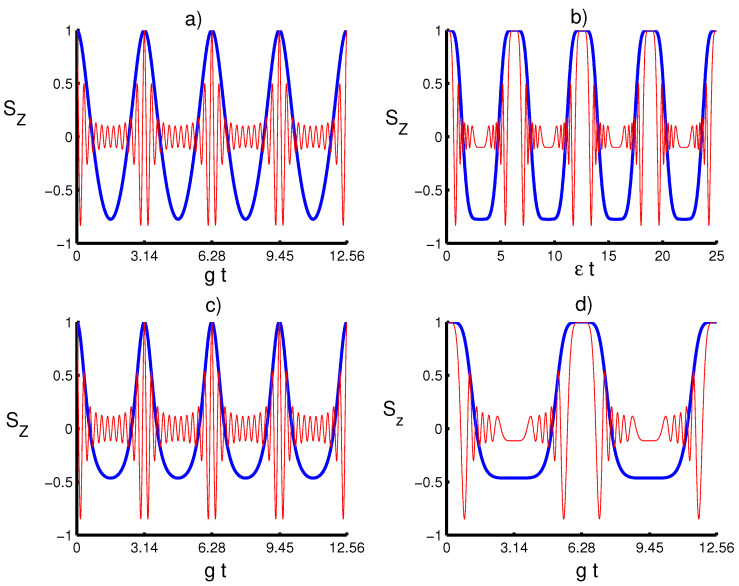
Population inversion for the two-level atom (TLA) initially defined in its excited state, and the field is in photon-added coherent states of the pseudoharmonic oscillator (PA-PHOCSs) for z=0.5. (**a**) The solid line (blue) is for $\left(k,m\right)=\left(\frac{1}{4},0\right)$, and the dashed line (red) is for $\left(k,m\right)=\left(\frac{1}{4},10\right)$. (**b**) The solid line (blue) is for $\left(k,m\right)=\left(\frac{1}{4},0\right)$, and the solid line (red) is for $\left(k,m\right)=\left(\frac{1}{4},10\right)$. (**c**) the solid line is for $\left(k,m\right)=\left(\frac{3}{4},0\right)$ and the dashed line $\left(k,m\right)=\left(\frac{3}{4},10\right)$. (**d**) The solid line (blue) is for $\left(k,m\right)=\left(\frac{3}{4},0\right)$ and the solid line (red) is for $\left(k,m\right)=\left(\frac{3}{4},10\right)$. In (**a**,**c**), we consider the case of constant coupling λ(t)=g and in (**b**,**d**), the time dependent coupling $\lambda \left(t\right)=g\sin^{2}\left(t\right)$.

In Figure 1, for a TLA initially defined in an upper-level state and the quantum field in PA-PHOCSs, we display the variation of the population inversion $S_{z}$ dimensionless for the scaled time gt with respect to various physical parameters. We compare the effects of parameters *k* and *m* in both cases with and without the time effect. We can see that the population inversion makes period oscillations during the time-evolution. The atomic inversion after suddenly decreasing to its minimum value at the beginning of the interaction increases to a maximum value for each periodicity in the case of m=0. When m≠0, the behavior of the atomic inversion makes rapid oscillations exhibiting local minima and local maxima in each periodicity. Moreover, as is seen, the parameter *k* has an impact on the temporal evolution of the atomic inversion only in the absence of photon excitation k=0 and leads to a decrease in the amount of $S_{z}$ by a suitable choice *k*. On the other hand, the existence of time-dependent coupling influence leads to a reduction of the oscillations of the $S_{z}$ during the time-evolution.

**Figure 2:**
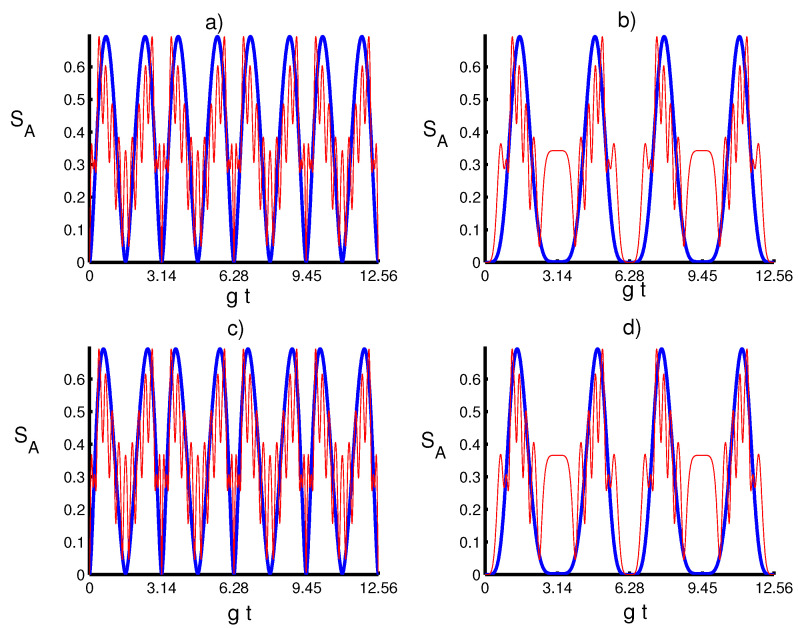
Von Neumann entropy $S_{A}$ with the same conditions of Figure 1. (**a**) The solid line (blue) is for $\left(k,m\right)=\left(\frac{1}{4},0\right)$, and the dashed line (red) is for $\left(k,m\right)=\left(\frac{1}{4},10\right)$. (**b**) The solid line (blue) is for $\left(k,m\right)=\left(\frac{1}{4},0\right)$, and the solid line (red) is for $\left(k,m\right)=\left(\frac{1}{4},10\right)$. (**c**) the solid line is for $\left(k,m\right)=\left(\frac{3}{4},0\right)$ and the dashed line $\left(k,m\right)=\left(\frac{3}{4},10\right)$. (**d**) The solid line (blue) is for $\left(k,m\right)=\left(\frac{3}{4},0\right)$ and the solid line (red) is for $\left(k,m\right)=\left(\frac{3}{4},10\right)$.

The numerical results of the von Neumann entropy have been shown versus the time gt in Figure 2 for various values of the photon-added number in the absence and existence of the time-dependent coupling influence when the TLA initially stated in the upper level and the quantum field is in PA-PHOCSs. The dashed line (red) is for m=0, and the solid line (blue) is for m=10. Figure 1c,d presents the temporal evolution of the entanglement for λ(t)=g and $\lambda \left(t\right)=g\sin^{2}\left(t\right)$, respectively, in the case of k=3/4. Generally, the von Neumann is a periodic function with sudden death and sudden birth entanglement phenomenon during the time-evolution. In the ideal case in which no atomic motion is considered, von Neumann entropy suddenly increases from zero to its maximum value, then it decays to zero for each periodic time interval, whereas when the time-dependent effect is considered, $S_{A}$ attains rapid oscillations due to the fluctuations during the interaction, but also leads to an enhancement or reduction of the degree of entanglement. This shows that the quantum field system can help to stabilize the temporal evolution of the entanglement. This behavior is due only to the influence of the kind of coupling term via the generalized parameter λ(t) and the photon-added number *m*. Moreover, it is found that as the parameter *m* increases, the structure of the oscillations becomes very complex for different values of *k*, whereas the coupling effect leads to the disappearance of these structures of the von Neumann entropy, i.e., the periodicity time increases and the oscillations are more transparent and accompanied by an increase in the lifetime of sudden death entanglement phenomenon. From Figure 1 and Figure 2, an interesting relationship can be seen between the dynamical behavior of the population inversion and the quantum entanglement.

**Figure 3:**
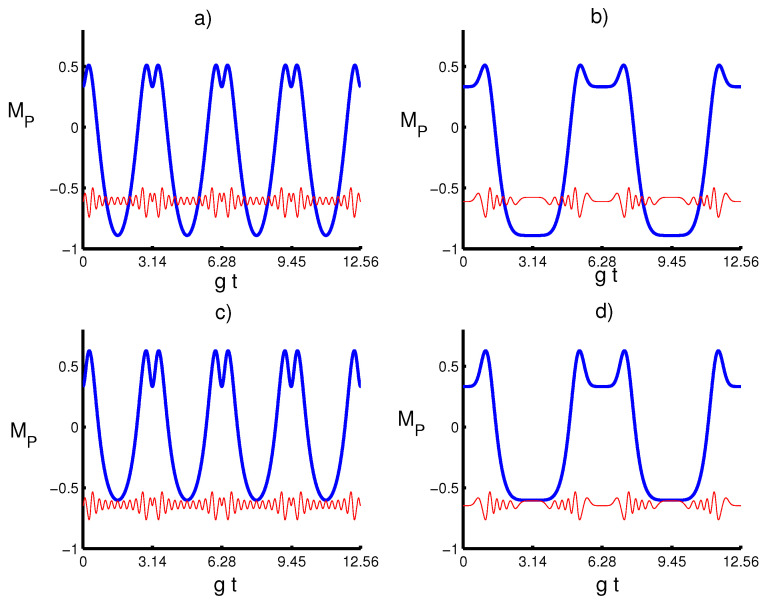
Parameter $M_{P}$ with the same situation as in Figure 1. (**a**) The solid line (blue) is for $\left(k,m\right)=\left(\frac{1}{4},0\right)$, and the dashed line (red) is for $\left(k,m\right)=\left(\frac{1}{4},10\right)$. (**b**) The solid line (blue) is for $\left(k,m\right)=\left(\frac{1}{4},0\right)$, and the solid line (red) is for $\left(k,m\right)=\left(\frac{1}{4},10\right)$. (**c**) the solid line is for $\left(k,m\right)=\left(\frac{3}{4},0\right)$ and the dashed line $\left(k,m\right)=\left(\frac{3}{4},10\right)$. (**d**) The solid line (blue) is for $\left(k,m\right)=\left(\frac{3}{4},0\right)$ and the solid line (red) is for $\left(k,m\right)=\left(\frac{3}{4},10\right)$.

Figure 3 refers to the effect of the parameters *m* and *k* on the time evolution of Mandel’s $M_{P}$ parameter defined by Equation (11) when the field is initially defined in PA-PHOCSs in the absence and existence of the time-dependent coupling influence. Generally, Mandel’s parameter makes periodic oscillations, exhibiting a sub-Poissonian and Poissonian distribution at m=0 for different values of *k*. Whereas, for m≠0, the field statistics tend to fluctuate around the sub-Poissonian distribution. Interestingly, we obtain that the choice of the initial parameter *k* only influences the photon statistics of the quantum field in the absence of the excited photons. When the time-dependent effect is considered, Mandel’s parameter keeps its behavior with an increase in the periodic time interval during the evolution.

**Figure 4:**
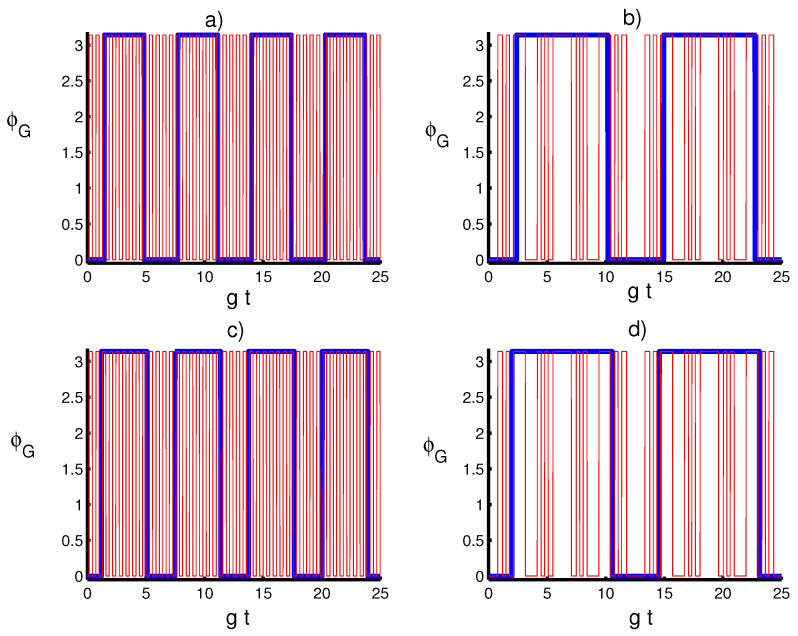
Geometric phase $\phi_{P}$ with the same situation as in Figure 1. (**a**) The solid line (blue) is for $\left(k,m\right)=\left(\frac{1}{4},0\right)$, and the dashed line (red) is for $\left(k,m\right)=\left(\frac{1}{4},10\right)$. (**b**) The solid line (blue) is for $\left(k,m\right)=\left(\frac{1}{4},0\right)$, and the solid line (red) is for $\left(k,m\right)=\left(\frac{1}{4},10\right)$. (**c**) the solid line is for $\left(k,m\right)=\left(\frac{3}{4},0\right)$ and the dashed line $\left(k,m\right)=\left(\frac{3}{4},10\right)$. (**d**) The solid line (blue) is for $\left(k,m\right)=\left(\frac{3}{4},0\right)$ and the solid line (red) is for $\left(k,m\right)=\left(\frac{3}{4},10\right)$.

Let us investigate the main results on the variation of the GP for the whole system state |ψ〉 with respect to the physical parameters in the presence and absence of the time-depending coupling influence. To understand the impact of the parameters’ effects on $\Phi_{G}$, we display the dynamical behavior of the $\Phi_{G}$ in Figure 4 with respect to different values of *k* and *m*. Generally, it can be seen that the GP provides a periodic behavior, exhibiting collapse and revival phenomena. The duration of these phenomena strictly depends on the excited number *m* and coupling term λ(t), where the atomic motion leads to an increase in the periodicity time of the GP. On the other hand, for large values of *m*, the GP is unaffected by the parameter *k*, and the result seems to be similar for both cases k=1/4 and k=3/4. From the obtained results, we find that the control and the stabilization of the system dynamics highly benefit from the combination of the quantum field and coupling term parameters.

**Figure 5:**
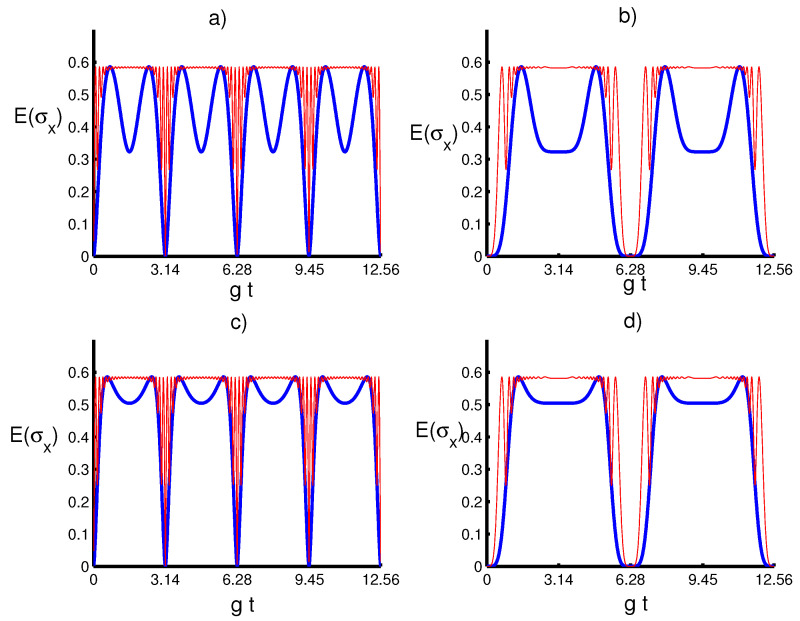
Entropy squeezing component $E\left(\sigma_{x}\right)$ with the same conditions of Figure 1. (**a**) The solid line (blue) is for $\left(k,m\right)=\left(\frac{1}{4},0\right)$, and the dashed line (red) is for $\left(k,m\right)=\left(\frac{1}{4},10\right)$. (**b**) The solid line (blue) is for $\left(k,m\right)=\left(\frac{1}{4},0\right)$, and the solid line (red) is for $\left(k,m\right)=\left(\frac{1}{4},10\right)$. (**c**) the solid line is for $\left(k,m\right)=\left(\frac{3}{4},0\right)$ and the dashed line $\left(k,m\right)=\left(\frac{3}{4},10\right)$. (**d**) The solid line (blue) is for $\left(k,m\right)=\left(\frac{3}{4},0\right)$ and the solid line (red) is for $\left(k,m\right)=\left(\frac{3}{4},10\right)$.

**Figure 6:**
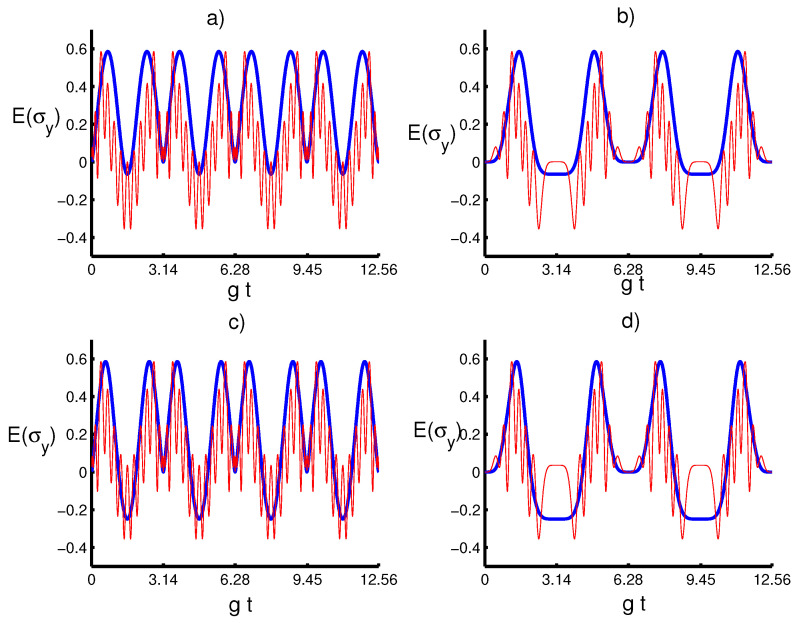
Entropy squeezing component $E\left(\sigma_{y}\right)$ with the same conditions of Figure 1. (**a**) The solid line (blue) is for $\left(k,m\right)=\left(\frac{1}{4},0\right)$, and the dashed line (red) is for $\left(k,m\right)=\left(\frac{1}{4},10\right)$. (**b**) The solid line (blue) is for $\left(k,m\right)=\left(\frac{1}{4},0\right)$, and the solid line (red) is for $\left(k,m\right)=\left(\frac{1}{4},10\right)$. (**c**) the solid line is for $\left(k,m\right)=\left(\frac{3}{4},0\right)$ and the dashed line $\left(k,m\right)=\left(\frac{3}{4},10\right)$. (**d**) The solid line (blue) is for $\left(k,m\right)=\left(\frac{3}{4},0\right)$ and the solid line (red) is for $\left(k,m\right)=\left(\frac{3}{4},10\right)$.

We now examine the dynamical behavior of the atomic squeezing with regard to the physical parameters. In Figure 5 and Figure 6, we plot the time-evolution of $E(\sigma_{x})$ and $E(\sigma_{y})$ versus the dimensionless time gt, respectively, with respect to different values of the parameters *m* and *k* for both cases λ(t)=g and $\lambda \left(t\right)=g\sin^{2}\left(t\right).$ We find that the atomic squeezing provides periodic oscillations, where $E(\sigma_{x})$ and $E(\sigma_{y})$ remain unchanged under the parameter *k* as the added photon number *m* obtains large values. This shows that the enhancement and loss of squeezing are due to the physical properties of the quantum field. Interestingly, the atomic motion leads to an increase in the time periodicity of the squeezing entropies. On the other hand, it seems that the squeezing occurs only in the variable *y* and no squeezing in *x*, where the increase in *m* would be accompanied by an increase in $E(\sigma_{y})$ and enhance the squeezing effect during the time-evolution. In a nutshell, the obtained results provide that the effect of the initial parameters *k* and *m* on the physical quantities seems to be the same in the existence and absence of the atomic motion influence, showing a monotonic relationship between these quantifiers with respect to the initial parameters.

## 5. Conclusions

We have developed a JC model considering the interaction between a TLA and a quantum field in the framework of pseudoharmonic oscillator potentials. We have shown the necessary optimal conditions that are appropriate for empirical implementation to execute various tasks of quantum computational and information technologies. We have examined qualitatively various quantum quantifiers in terms of the initial parameters during the time-evolution with and without time-dependent coupling, considering the quantum entanglement, geometric phase, nonclassicality and atomic squeezing. Furthermore, we have displayed the relationship between the different physical quantities in terms of the initial parameters during the evolution. We have shown that the change of the parameters strongly influences the dynamical behavior of the quantifiers. The obtained results confirm that the considered quantum system is helpful to withstand the effect of noises on the physical quantities by a suitable choice of the initial parameters. The result suggests future study, considering that the initial mixed state under the effect of the finite-temperature environments on the quantifiers could be pondered.

## References

[1] Nielsen M.A., Chuang I.L. (2000). Quantum Computation and Information.

[2] Alber G., Beth T., Horodecki M., Horodecki P., Horodecki R., Rötteler M., Weinfurter H., Zeilinger R.A. (2001). Quantum Information.

[3] Horodecki R., Horodecki P., Horodecki M., Horodecki K. (2009). Quantum entanglement. Rev. Mod. Phys..

[4] Joo J., Munro W.J., Spiller T.P. (2011). Quantum metrology with entangled coherent states. Phys. Rev. Lett..

[5] Amico L., Fazio R., Osterloh A., Vedral V. (2008). Entanglement in many-body systems. Rev. Mod. Phys..

[6] Gershenfeld N.A., Chuang I.L. (1997). Bulk spin-resonance quantum computation. Science.

[7] Tóth G., Simon C., Cirac J.I. (2003). Entanglement detection based on interference and particle counting. Phys. Rev. A.

[8] Sete E.A., Eleuch H., Ooi C.R. (2014). Light-to-matter entanglement transfer in optomechanics. JOSA B.

[9] Zheng S.B., Guo G.C. (2000). Efficient scheme for two-atom entanglement and quantum information processing in cavity QED. Phys. Rev. Lett..

[10] Blinov B.B., Moehring D.L., Duan L.M., Monroe C. (2004). Observation of entanglement between a single trapped atom and a single photon. Nature.

[11] Togan E., Chu Y., Trifonov A.S., Jiang L., Maze J., Childress L., Dutt M.G., Sørensen A.S., Hemmer P.R., Zibrov A.S. (2010). Quantum entanglement between an optical photon and a solid-state spin qubit. Nature.

[12] Wilk T., Webster S.C., Kuhn A., Rempe G. (2007). Single-atom single-photon quantum interface. Science.

[13] Olmschenk S., Matsukevich D.N., Maunz P., Hayes D., Duan L.M., Monroe C. (2009). Quantum teleportation between distant matter qubits. Science.

[14] Yuan Z.S., Chen Y.A., Zhao B., Chen S., Schmiedmayer J., Pan J.W. (2008). Experimental demonstration of a BDCZ quantum repeater node. Nature.

[15] Ritter S., Nölleke C., Hahn C., Reiserer A., Neuzner A., Uphoff M., Mücke M., Figueroa E., Bochmann J., Rempe G. (2012). An elementary quantum network of single atoms in optical cavities. Nature.

[16] Berry M.V. (1984). Quantal phase factors accompanying adiabatic changes. Proc. R. Soc. Lond. A.

[17] Aharonov Y., Anandan J. (1987). Phase change during a cyclic quantum evolution. Phys. Rev. Lett..

[18] Simon B. (1983). Holonomy, the quantum adiabatic theorem, and Berry’s phase. Phys. Rev. Lett..

[19] Anandan J., Stodolsky L. (1987). Some geometrical considerations of Berry’s phase. Phys. Rev. D.

[20] Samuel J., Bhandari R. (1988). General setting for Berry’s phase. Phys. Rev. Lett..

[21] Liu T., Cao X.Z., Su Q.P., Xiong S.J., Yang C.P. (2016). Multi-target-qubit unconventional geometric phase gate in a multi-cavity system. Sci. Rep..

[22] Feng X.L., Wang Z., Wu C., Kwek L.C., Lai C.H., Oh C.H. (2007). Scheme for unconventional geometric quantum computation in cavity QED. Phys. Rev. A.

[23] Xiang-Bin W., Keiji M. (2001). Nonadiabatic conditional geometric phase shift with NMR. Phys. Rev. Lett..

[24] Zhu S.L., Wang Z.D. (2002). Implementation of universal quantum gates based on nonadiabatic geometric phases. Phys. Rev. Lett..

[25] Falci G., Fazio R., Palma G.M., Siewert J., Vedral V. (2000). Detection of geometric phases in superconducting nanocircuits. Nature.

[26] Drummond P.D., Ficek Z. (2004). Quantum Squeezing.

[27] Wodkiewicz K. (1985). Reduced quantum fluctuations in the Josephson junction. Phys. Rev. B.

[28] Agarwal G.S., Puri R.R. (1990). Cooperative behavior of atoms irradiated by broadband squeezed light. Phys. Rev. A.

[29] Ashraf M.M., Razmi M.S.K. (1992). Atomic-dipole squeezing and emission spectra of the nondegenerate two-photon Jaynes-Cummings model. Phys. Rev. A.

[30] Kitagawa M., Ueda M. (1993). Squeezed spin states. Phys. Rev. A.

[31] Civitarese O., Reboiro M. (2006). Atomic squeezing in three level atoms. Phys. Lett. A.

[32] Civitarese O., Reboiro M., Rebón L., Tielas D. (2010). Atomic squeezing in three-level atoms with effective dipole–dipole atomic interaction. Phys. Lett. A.

[33] Poulsen U.V., Mølmer K. (2001). Squeezed light from spin-squeezed atoms. Phys. Rev. Lett..

[34] Yukalov V.I., Yukalova E.P. (2004). Atomic squeezing under collective emission. Phys. Rev. A.

[35] Wang X. (2001). Spin squeezing in nonlinear spin-coherent states. J. Opt. B Quantum Semiclass. Opt..

[36] Rojo A.G. (2003). Optimally squeezed spin states. Phys. Rev. A.

[37] Wang X., Sanders B.C. (2003). Relations between bosonic quadrature squeezing and atomic spin squeezing. Phys. Rev. A.

[38] Dicke R.H. (1954). Coherence in spontaneous radiation processes. Phys. Rev..

[39] El-Orany F.A., Wahiddin M.R.B., Obada A.S. (2008). Single-atom entropy squeezing for two two-level atoms interacting with a single-mode radiation field. Opt. Commun..

[40] Barnett S.M., Knight P.L. (1986). Dissipation in a fundamental model of quantum optical resonance. Phys. Rev. A.

[41] Puri R.R., Agarwal G.S. (1987). Finite-Q cavity electrodynamics: Dynamical and statistical aspects. Phys. Rev. A.

[42] Eiselt J., Risken H. (1991). Quasiprobability distributions for the Jaynes-Cummings model with cavity damping. Phys. Rev. A.

[43] Abdel-Khalek S., Obada A.S. (2010). New features of Wehrl entropy and Wehrl PD of a single Cooper-pair box placed inside a dissipative cavity. Ann. Phys..

[44] Klauder J.R., Skagerstam B.S. (1985). Coherent States-Applications in Physics and Mathematical Physics.

[45] Zhang W.M., Gilmore R. (1990). Coherent states: Theory and some applications. Rev. Mod. Phys..

[46] Glauber R.J. (1963). The quantum theory of optical coherence. Phys. Rev..

[47] Walls D.F. (1983). Squeezed states of light. Nature.

[48] Loudon R., Knight P.L. (1987). Squeezed light. J. Mod. Opt..

[49] Popov D. (2001). Barut-Girardello coherent states of the pseudoharmonic oscillator. J. Phys. A Math. Gen..

[50] Popov D., Sajfert V., Zaharie I. (2008). Pseudoharmonic oscillator and their associated Gazeau–Klauder coherent states. Phys. A Stat. Mech. Appl..

[51] Sage M., Goodisman J. (1985). Improving on the conventional presentation of molecular vibrations: Advantages of the pseudoharmonic potential and the direct construction of potential energy curves. Am. J. Phys..

[52] Gazeau J.P., Klauder J.R. (1999). Coherent states for systems with discrete and continuous spectrum. J. Phys. A Math. Gen..

[53] Klauder J.R., Penson K.A., Sixdeniers J.M. (2001). Constructing coherent states through solutions of Stieltjes and Hausdorff moment problems. Phys. Rev. A.

[54] Gol’dman I.I., Krivchenko V.D., Kogan V.I., Galitskiy V.M. (1960). Problems in Quantum Mechanics.

[55] Roy B., Roy P. (2002). Gazeau–Klauder coherent state for the Morse potential and some of its properties. Phys. Lett. A.

[56] Fakhri H., Chenaghlou A. (2003). Barut–Girardello coherent states for the Morse potential. Phys. Lett. A.

[57] Popov D. (2003). Gazeau–Klauder quasi-coherent states for the Morse oscillator. Phys. Lett. A.

[58] Popov D., Davidovic D.M., Arsenovic D., Saifert V. (2006). P-function of the pseudo harmonic oscillator in terms of Klauder-Perelomov coherent states. Acta Phys. Slovaca.

[59] Walls D.F., Milburn G.J. (1994). Quantum Optics.

[60] Wang X., Sanders B.C., Pan S.H. (2000). Entangled coherent states for systems with SU (2) and SU (1, 1) symmetries. J. Phys. A Math. Gen..

[61] Popov D., Pop N., Luminosu I., Chiriţoiu V. (2009). Density matrix approach of the excitation on coherent states of the pseudoharmonic oscillator. EPL Europhys. Lett..

[62] Mojaveri B., Dehghani A. (2013). Generalized su (1, 1) coherent states for pseudo harmonic oscillator and their nonclassical properties. Eur. Phys. J. D.

[63] Perelomov A.M. (1972). Coherent states for arbitrary Lie group. Commun. Math. Phys..

[64] Wodkiewicz K., Eberly J.H. (1985). Coherent states, squeezed fluctuations, and the SU (2) am SU (1, 1) groups in quantum-optics applications. JOSA B.

[65] Popov D., Pop N., Sajfert V. (2009). Excitation on the Coherent States of Pseudoharmonic Oscillator. AIP Conf. Proc..

[66] Perelomov A.M. (1986). Generalized Coherent States and Their Applications.

[67] Gerry C.C., Silverman S. (1982). Path integral for coherent states of the dynamical group SU (1, 1). J. Math. Phys..

[68] Janes E.T., Cummings F.W. (1963). Comparison of quantum and semiclassical radiation theories with application to the beam maser. Proc. IEEE.

[69] Phoenix S.J., Knight P.L. (1991). Comment on “Collapse and revival of the state vector in the Jaynes-Cummings model: An example of state preparation by a quantum apparatus”. Phys. Rev. Lett..

[70] Friedrich B., Herschbach D. (1995). Alignment and trapping of molecules in intense laser fields. Phys. Rev. Lett..

[71] Rempe G., Walther H., Klein N. (1987). Observation of quantum collapse and revival in a one-atom maser. Phys. Rev. Lett..

[72] Scully M.O., Zubairy M.S. (1997). Quantum Optics.

[73] Von Neumann J. (1955). Mathematical Foundations of Quantum Mechanics.

[74] Berrada K., El Baz M., Hassouni Y. (2011). On the construction of generalized su (1, 1) coherent states. Rep. Math. Phys..

[75] Abdel-Khalek S., Berrada K., Ooi C.R. (2012). Beam splitter entangler for nonlinear bosonic fields. Laser Phys..

[76] Pancharatnam S. (1956). The adiabatic phase and pancharatnam’s phase for polarized light. Proc. Indian Acad. Sci..

[77] Fang M.F., Zhou P., Swain S. (2000). Entropy squeezing for a two-level atom. J. Mod. Opt..

